# Development of the Periventricular Nucleus as a Brain Center, Containing Dopaminergic Neurons and Neurons Expressing Individual Enzymes of Dopamine Synthesis

**DOI:** 10.3390/ijms232314682

**Published:** 2022-11-24

**Authors:** Tatiana Pronina, Ekaterina Pavlova, Liliya Dil’mukhametova, Michael Ugrumov

**Affiliations:** Laboratory of Neural and Neuroendocrine Regulations, Koltzov Institute of Developmental Biology of the Russian Academy of Sciences, 119334 Moscow, Russia

**Keywords:** brain, periventricular nucleus, dopaminergic neuron, dopamine, L-DOPA, tyrosine hydroxylase, aromatic L-amino acid decarboxylase, 3rd cerebral ventricle, rat, ontogenesis

## Abstract

We have recently shown that the periventricular nucleus (PeVN) of adult rats is a “mixed dopaminergic (DAergic) center” containing three thousand neurons: DAergic neurons and those expressing one of the dopamine (DA)-synthesizing enzymes. This study aims to evaluate the development of the PeVN as a mixed DAergic center in rats in the perinatal period, critical for brain morphogenesis. During this period, the PeVN contains DAergic neurons and monoenzymatic neurons expressing individual enzymes of DA synthesis: tyrosine hydroxylase (TH) or aromatic L-amino acid decarboxylase (AADC). In the perinatal period, the total number of such neurons triples, mainly due to monoenzymatic neurons; the content of L-DOPA, the end product of monoenzymatic TH neurons, doubles; and the content of DA, the end product of monoenzymatic AADC neurons and DAergic neurons, increases sixfold. Confocal microscopy has shown that, in the PeVN, all types of neurons and their processes are in close relationships, which suggests their mutual regulation by L-DOPA and DA. In addition, monoenzymatic and DAergic fibers are close to the third cerebral ventricle, located in the subependymal zone, between ependymal cells and in the supraependymal zone. These observations suggest that these fibers deliver L-DOPA and DA to the cerebrospinal fluid, participating in the neuroendocrine regulation of the brain.

## 1. Introduction

With the development, mainly in the second half of the last century, of methods for detecting monoamines, visualizing monoaminergic neurons, and evaluating the mechanisms of monoamine action, it has been repeatedly shown that monoamines in adulthood are synthesized everywhere in the brain, participating as neurotransmitters or neuromodulators in the regulation of numerous functions of the brain and the body as a whole [1,2,3]. The most widely distributed and functionally important monoamines are norepinephrine, dopamine (DA), and serotonin [2].

Thanks to the histofluorescent method for the detection of monoamines first, and the immunohistochemical method for the detection of key enzymes of monoamine synthesis later, it has been possible to map large clusters of these neurons in the brain, designated as monoaminergic centers [4,5,6]. The emergence of new opportunities for the immunocytochemical detection of a wider range of protein markers of monoaminergic neurons has confirmed the initial idea that the raphe nucleus contains serotonergic neurons, and that the locus coeruleus contains norepinephrinergic neurons [5,7,8]. With regard to dopaminergic (DAergic) centers, which were initially identified biochemically by the high content of DA, and immunocytochemically by the presence of numerous neurons expressing tyrosine hydroxylase (TH), the first enzyme of DA synthesis [5,9], the situation turned out to be more complicated. Indeed, when using double immunolabeling for TH and aromatic L-amino acid decarboxylase (AADC), the second enzyme of DA synthesis, it has been shown that some DAergic centers, for example, the compact part of the substantia nigra, contain practically only DAergic (bienzymatic) neurons, whereas in other DAergic centers, along with DAergic neurons, there are neurons expressing only one of the DA-synthesizing enzymes, TH or AADC [3,10]. Such “mixed” DAergic centers include: the arcuate nucleus (AN), the striatum, the periventricular nucleus (PeVN), and a number of other smaller centers [3,11].

Studies following the discovery of neurons containing one of the DA-synthesizing enzymes have shown that: (i) the number of monoenzymatic neurons in the brain exceeds the number of DAergic neurons [3,12,13,14,15,16,17]; (ii) the end secretory product of neurons expressing only TH is L-3,4-dihydroxyphenylalanine (L-DOPA), and the end product of neurons expressing only AADC, or TH and AADC, is DA [18,19,20]; (iii) neurons expressing only TH and only AADC synthesize DA in cooperation [20]; (iv) cooperative synthesis of DA by monoenzymatic neurons is considered as a compensatory response to a failure of DAergic neurons and the resulting DA deficiency, e.g., due to neurodegeneration [21,22]; and (v) L-DOPA and DA synthesized by monoenzymatic neurons play the role of neurotransmitters in the nervous tissue or that of a neurohormone when delivered into the cerebrospinal fluid or blood [11,23,24,25]. The above data were obtained mainly from the study of the AN, which was identified already in the early 2000s as a mixed DAergic center [26,27]. After that, at least two large mixed DAergic centers were identified, the striatum and the PeVN, which have recently attracted particular attention of researchers [11,22].

The study of the development and functional role of mixed DAergic centers in ontogenesis is of particular importance for developmental biology and neurophysiology, since in the perinatal period their end secretory products L-DOPA and DA can potentially participate as morphogenetic factors in the regulation of brain development. Our extensive experience in studying the development of the AN as a mixed DAergic center, accumulated over the past 20 years, has shown that such research should focus on studying the formation of these centers in the perinatal period [3,28]. Indeed, it is at this time that the differentiation of neurons and the formation of specific neuronal systems in the brain occur under the influence of a wide range of morphogenetic factors: monoamines, neuropeptides, steroid hormones, and others [29,30,31]. It is also noteworthy that monoenzymatic neurons become functionally active during this period of ontogenesis. In fact, studies of the development of the AN have shown that DA inhibitory control of pituitary prolactin secretion, characteristic of adult animals, begins in rats at the end of the prenatal period, when this mixed DAergic center contains only monoenzymatic neurons [3,28,32]. As the AN develops in the perinatal period, the proportion of DAergic neurons gradually increases in relation to monoenzymatic neurons [26]. At the same time, the role of DAergic neurons in the inhibitory control of prolactin secretion increases [23].

The aim of this study has been to continue examining the development of mixed DAergic centers of the hypothalamus in ontogenesis. This is the first study of the development of PeVN as a mixed DAergic center.

## 2. Results

### 2.1. Dopamine and L-DOPA Levels in the Periventricular and Arcuate Nuclei in Perinatal Rats

**Figure 1:**
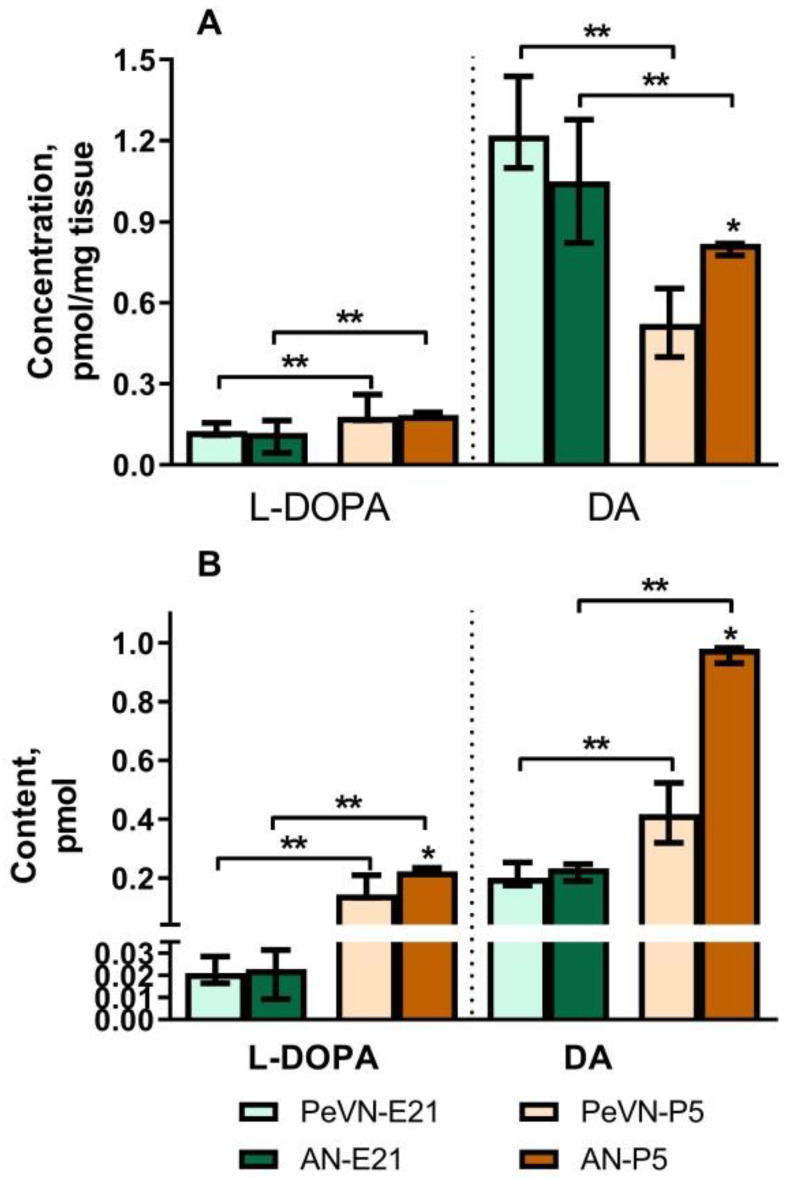
Concentration (**A**) and content (**B**) of dopamine (DA) and L-3,4-dihydroxyphenylalanine (L-DOPA) in the periventricular nucleus (PeVN) and in the arcuate nucleus (AN) on embryonic day 21 (E21) and on postnatal day 5 (P5). * *p* < 0.05, significant differences in the concentration and content of DA in the PeVN and AN; ** *p* < 0.05, significant differences between the selected parameters (unpaired Wilcoxon test). Data are presented as median with interquartile range.

On embryonic day 18 (E18), DA and L-DOPA were not detected in the PeVN. On E21, the concentration and content of DA and L-DOPA in the PeVN and AN do not differ. However, the concentration and content of DA in both nuclei is 10 times higher than the concentration and content of L-DOPA (Figure 1).

On P5, the concentration and content of DA in the PeVN are 36% and 57% lower than in the AN, respectively. In contrast to DA, the concentration and content of L-DOPA in the PeVN and AN do not differ in this age group. Interestingly, the concentration of DA in the PeVN on P5 is approximately 57% lower than in the same nucleus on E21, and the content is twice as high (Figure 1).

### 2.2. Dopamine Content in Vibratome Sections of the Periventricular Nucleus and Substantia Nigra in Rats on the 5th Postnatal Day, as Well as in the Incubation Medium after Incubating Sections in the Medium with or without 2-Aminobicyclo[2.2.1]heptane-2-Carboxylic Acid

**Figure 2:**
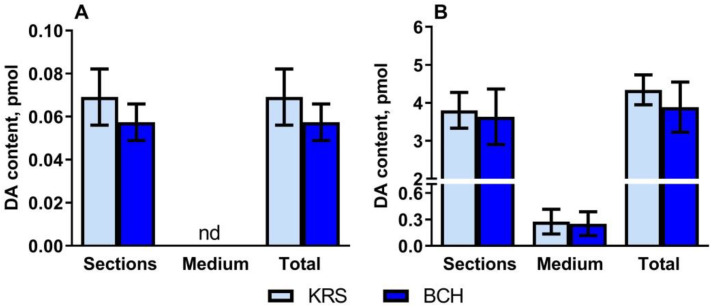
Dopamine content (DA): (i) in vibratome sections of the periventricular nucleus (**A**) or substantia nigra (**B**) of rats on postnatal day 5; (ii) in the medium after incubation of vibratome sections in Krebs-Ringer solution (KRS) with or without 0.5 mM 2-aminobicyclo[2.2.1]heptane-2-carboxylic acid (BCH); (iii) total in vibratome sections of the periventricular nucleus or substantia nigra and in the medium after their incubation of vibratome sections in KRS with or without BCH (paired *t*-test). Data are presented as mean ± SEM.

*Periventricular nucleus.* On P5, when PeVN vibratome sections were incubated in Krebs-Ringer solution (KRS), DA content was detected in the tissue but not in the incubation medium (Figure 2A). The addition of 0.5 mM 2-aminobicyclo[2.2.1]heptane-2-carboxylic acid (BCH) to the incubation medium did not change the content of DA in the tissue. DA was not detected in the medium (Figure 2A).

*Substantia nigra.* After incubation of vibratome sections of the substantia nigra (SN) of rats at P5 in KRS, the content of DA in the sections was 14 times higher than in the incubation medium (Figure 2B). The addition of BCH to the incubation medium did not change the DA content in either the sections or the incubation medium (Figure 2B).

### 2.3. The Number of Neurons Immunopositive for Tyrosine Hydroxylase, for Aromatic L-amino Acid Decarboxylase, and for Both Enzymes in the Periventricular Nucleus in Rats on the 18th and 21st Embryonic Days and on the 5th Postnatal Day

**Figure 3:**
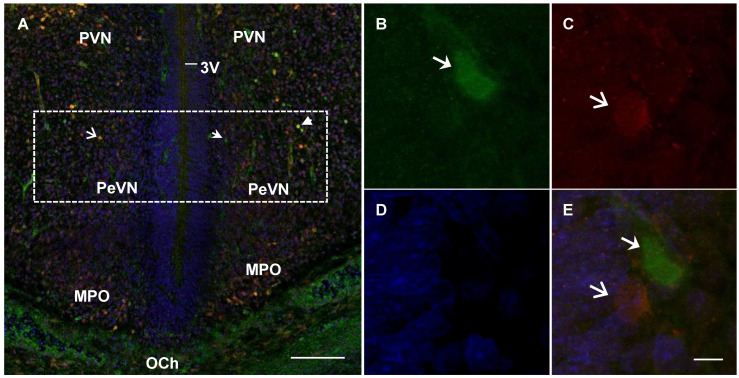
Confocal microscopy of the periventricular nucleus (PeVN) (frontal sections) of the rat on embryonic day 18. (**A**), general view of the PeVN (dotted frame) with sparse neurons (arrows), weakly immunopositive for either tyrosine hydroxylase (TH, green) or aromatic L-amino acid decarboxylase (AADC) (red). (**A**,**B**,**E**), neurons, immunopositive for TH, but immunonegative for AADC (green, arrow); (**A**,**C**,**E**), immunopositive for AADC, but immunonegative for TH (red, open arrow); (**A**), immunopositive for both enzymes (yellow, arrowhead). (**D**), cells with nuclei stained with DAPI (blue). 3V, 3rd cerebral ventricle; MPO, medial preoptic nucleus; OCh, optic chiasma; PVN, paraventricular nucleus. Bar: (**A**)—100 µm, (**B**–**E**)—5 µm.

**Figure 4:**
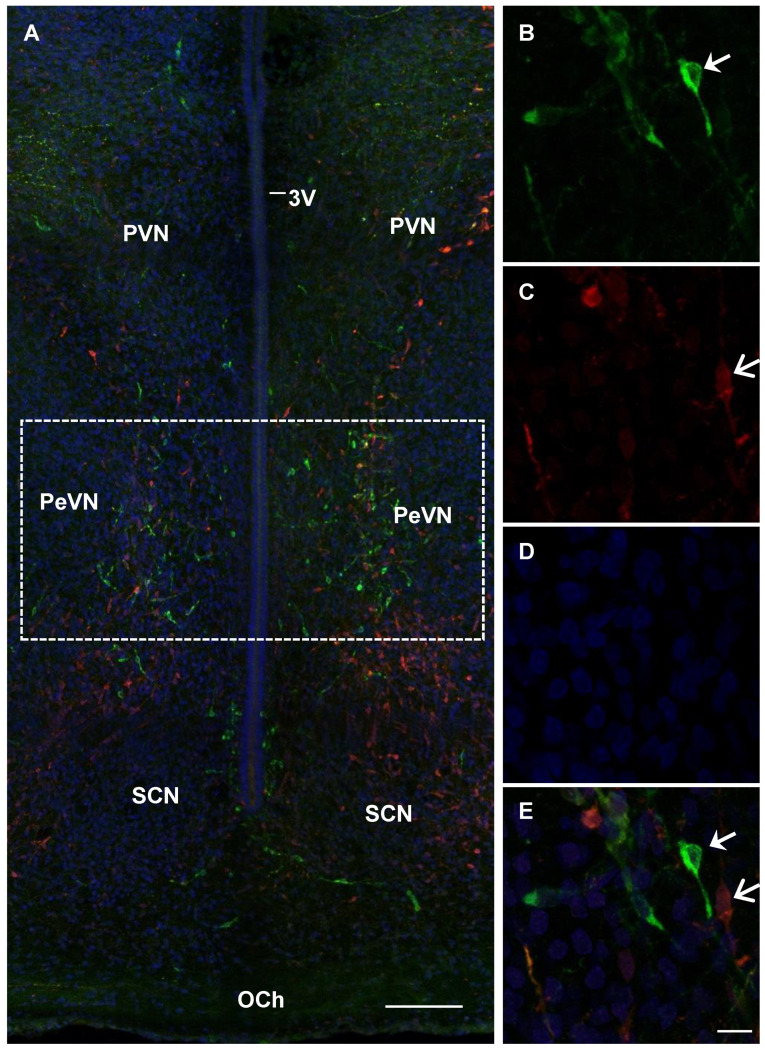
Confocal microscopy of the periventricular nucleus (PeVN) (frontal sections) of the rat on embryonic day 21. (**A**), general view of the PeVN (dotted frame) with neurons, immunopositive for either tyrosine hydroxylase (TH, green) or aromatic L-amino acid decarboxylase (AADC) (red). (**A**,**B**,**E**), neurons, immunopositive for TH, but immunonegative for AADC (green, arrow); (**C**,**E**), immunopositive for AADC, but immunonegative for TH (red, open arrow); (**A**), immunopositive for both enzymes (yellow). (**D**), cells with nuclei stained with DAPI (blue). 3V, 3rd cerebral ventricle; OCh, optic chiasma; PVN, paraventricular nucleus; SCN, suprachiasmatic nucleus. Bar: A—100 µm, (**B**–**E**)—10 µm.

**Figure 5:**
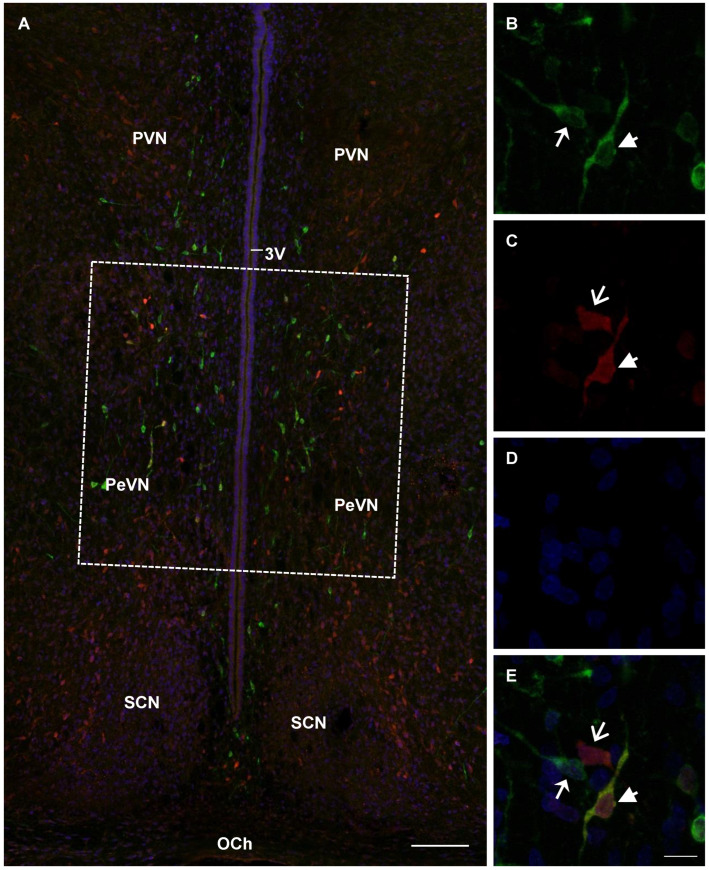
Confocal microscopy of the periventricular nucleus (PeVN) (frontal sections) of the rat on postnatal day 5. (**A**), general view of the PeVN (dotted frame) with neurons, immunopositive for either tyrosine hydroxylase (TH, green) or aromatic L-amino acid decarboxylase (AADC) (red). (**A**,**B**,**E**), neurons, immunopositive for TH, but immunonegative for AADC (green, arrow); (**C**,**E**), immunopositive for AADC, but immunonegative for TH (red, open arrow); (**B**,**C**,**E**), immunopositive for both enzymes (yellow, arrowhead). (**D**), cells with nuclei stained with DAPI (blue). 3V, 3rd cerebral ventricle; OCh, optic chiasma; PVN, paraventricular nucleus; SCN, suprachiasmatic nucleus. Bar: (**A**)—100 µm, (**B**–**E**)—10 µm.

In rats in all studied age groups (E18, E21, and P5), PeVN contained neurons immunopositive only for TH, immunopositive only for AADC, and immunopositive for both enzymes (Figure 3, Figure 4 and Figure 5).

**Figure 6:**
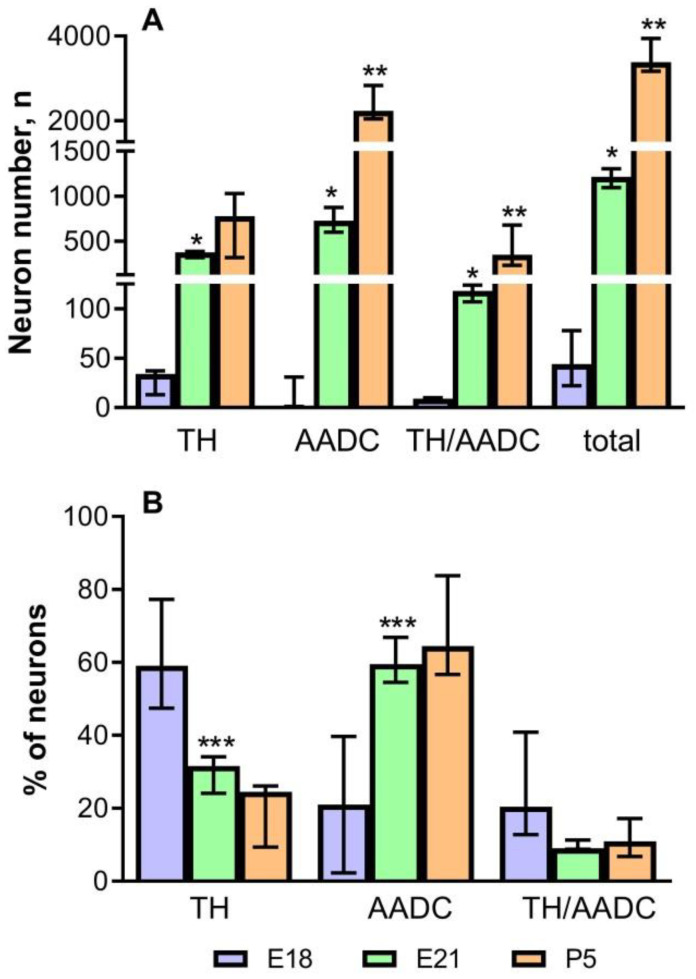
The total number of neurons immunopositive only for tyrosine hydroxylase (TH), only for aromatic L-amino acid decarboxylase (AADC) and for both dopamine-synthesizing enzymes (TH/AADC) (**A**), and their proportions (in %) of the total number of neurons that are immunopositive for dopamine-synthesizing enzymes (**B**), in the periventricular nucleus in rats on embryonic day (E18), on E21 and on postnatal day 5 (P5). * *p* < 0.05, significant differences between the total number of neurons immunopositive for the same dopamine-synthesizing enzymes on E18 and E21; ** *p* < 0.05, significant differences between the total number of neurons immunopositive for the same dopamine-synthesizing enzymes on E21 and P5; *** *p* < 0.05, significant differences between the proportions of neurons (in %), immunopositive for the same dopamine-synthesizing enzymes, at E18 and E21 (unpaired Wilcoxon test). Data are presented as median with interquartile range.

In rats of all age groups—on E18, E21, and P5—we calculated the total number of all neurons immunopositive for DA-synthesizing enzymes, as well as the number of neurons immunopositive only for TH, immunopositive only for AADC, and immunopositive for TH and AADC (Figure 6A). The total number of neurons immunopositive for DA-synthesizing enzymes in the PeVN on E18 is extremely small: about 50. Among them, the most numerous group (58.3%) are neurons immunopositive only for TH, while insignificant proportions of monoenzymatic AADC neuros (22.2%) and bienzymatic neurons (19.5%) differ little from each other (Figure 6B).

On E21, the total number of neurons immunopositive for DA-synthesizing enzymes averaged 1206 (Figure 6A). This age group is dominated by monoenzymatic neurons immunopositive for AADC (60.7%). Neurons immunopositive for TH only make up about 29.7%, and those immunopositive for both enzymes constitute 9.6% (Figure 6B).

On P5, the total number of neurons immunopositive for TH and AADC averaged 3497, i.e., almost 3 times as many as on E21 (Figure 6A). As on E21, neurons that are immunopositive only for AADC quantitatively dominate (67.8%) on P5, while the proportions of other neuron populations are much smaller: 20.3% for monoenzymatic TH neurons and 9.6% for bienzymatic neurons, respectively (Figure 6B).

**Figure 7:**
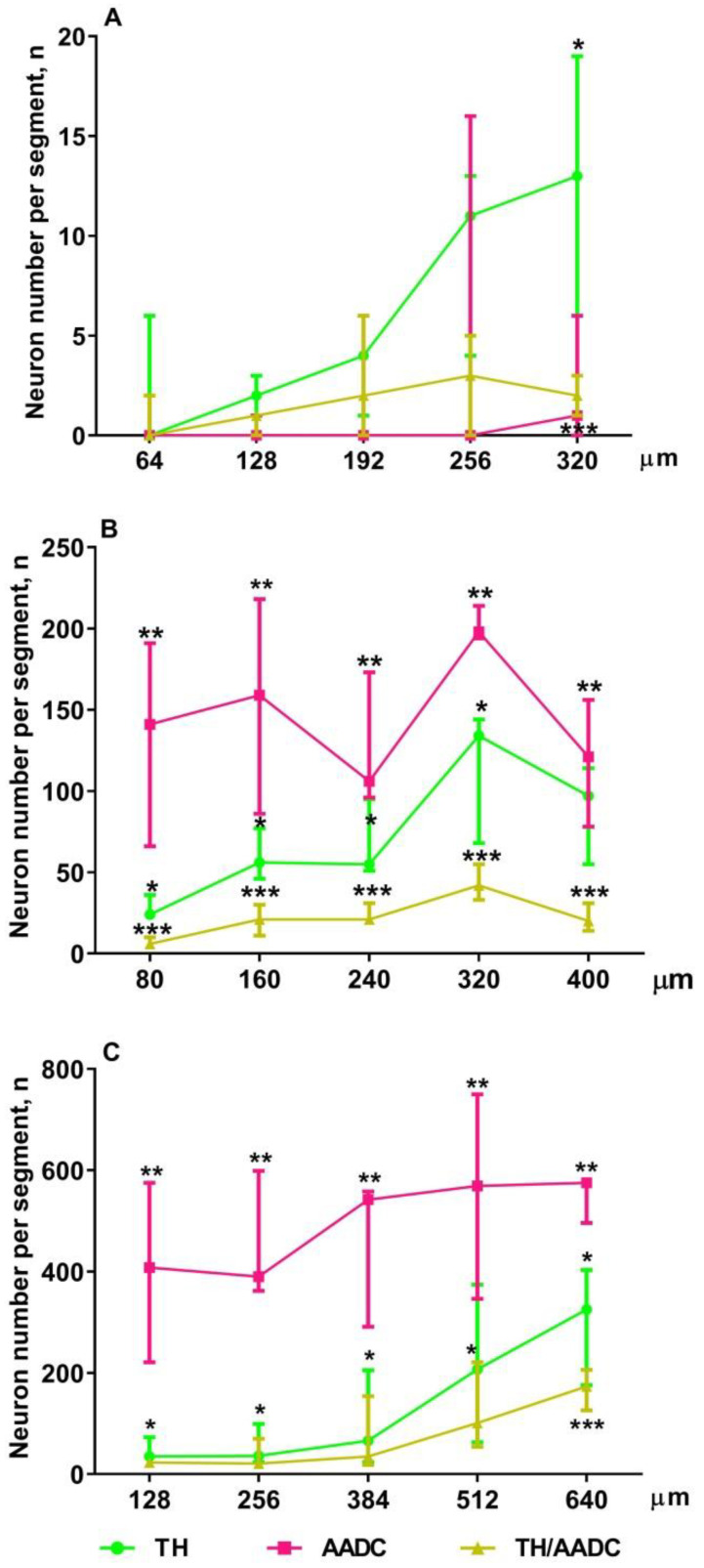
The number of neurons immunopositive only for tyrosine hydroxylase (TH), only for aromatic L-amino acid decarboxylase (AADC) and for both dopamine-synthesizing enzymes (TH/AADC) in five segments of the periventricular nucleus (PEN) of the same extent in the rostro-caudal direction in rats of each age: 64 µm on embryonic day 18 (E18) (**A**), 80 µm on E21 (**B**) and 128 µm on postnatal day 5 (P5) (**C**). * *p* < 0.05, significant difference between the number of TH neurons and AADC neurons contained in the same PeVN segment; ** *p* < 0.05, significant differences between the number of AADC-neurons and TH/AADC-neurons contained in the same PeVN segment; *** *p* < 0.05, significant difference between the number of TH/AADC-neurons and TH-neurons contained in the same PeVN segment (unpaired Wilcoxon test and Friedman test with Dunn’s post hoc test). Data are presented as median with interquartile range.

In addition to counting the total number of neurons immunopositive for DA-synthesizing enzymes, a quantitative analysis of the distribution of these neurons in the PeVN in the rostrocaudal direction was carried out. To do this, neurons were counted in successive conventionally identified PeVN segments in the rostrocaudal direction. On E18, the first two rostral segments contained only single neurons of all types (Figure 7A). In the more caudal segments 4 and 5, the number of neurons immunopositive for DA-synthesizing enzymes increased, but only slightly. The most numerous group was constituted by neurons immunopositive only for TH. In the caudal segments (4, 5), the number of TH neurons increased compared with the rostral segments (1–3). In the 5th caudal segment, the number of TH neurons was significantly higher compared to other types of neurons. The number of AADC neurons and bienzymatic neurons did not differ in different PeVN segments in the rostrocaudal direction (Figure 7A).

On E21, the number of all types of neurons in all PeVN segments in the rostrocaudal direction increased compared with E18 (Figure 7B). Neurons immunopositive only for AADC were most numerous and most evenly distributed over the segments. The smallest population was represented by neurons immunopositive for both enzymes. The number of these neurons per segment slightly increased in the rostrocaudal direction up to the 5th segment (Figure 7B). The population of neurons immunopositive only for TH also gradually increased in the caudal direction up to the 5th segment, but the number of these neurons in each segment was intermediate between the number of neurons immunopositive only for AADC and that of neurons immunopositive for both enzymes (Figure 7B).

On P5, the number of AADC-immunopositive neurons in all PeVN segments more than doubled, but their distribution in the rostrocaudal direction remained uniform (Figure 7C). The number of neurons immunopositive for TH and for both DA-synthesizing enzymes increased along the PeVN segments in the rostrocaudal direction, but not as significantly as the number of neurons immunopositive for AADC. The number of TH-immunopositive neurons slowly increased in the rostrocaudal direction, including the last 5th segment (Figure 7C).

### 2.4. Confocal Microscopy of Topographic Relationships of Neurons and Nerve Fibers, Immunopositive Only for Tyrosine Hydroxylase, Only for Aromatic L-amino acid Decarboxylase, and for Both Enzymes, with the 3rd Cerebral Ventricle in Rats on Embryonic Days 18 and 21 and on Postnatal Day 5

**Figure 8:**
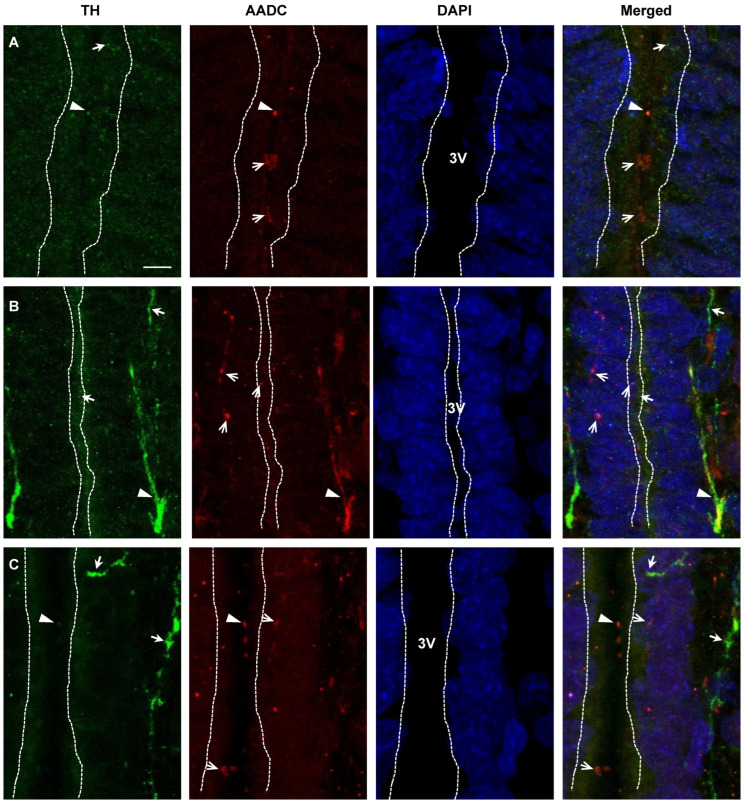
Confocal microscopy of the periventricular nucleus of rats on embryonic day 18 (**A**), embryonic day 21 (**B**) and postnatal day 5 (**C**): topographic relationships between the 3rd cerebral ventricle (3V, dotted line) and neurons (cell bodies and nerve fibers), immunopositive only for tyrosine hydroxylase (TH) (green, thick arrow), only for aromatic L-amino acid decarboxylase (AADC), (red, open arrow), and for both enzymes (TH + AADC, merged) (yellow, arrowhead). Fibers of all types are located in the subependymal zone, between the ependymal cells and in the 3rd ventricle. Cell nuclei are stained with DAPI (blue). Bar: 5 µm; ×63.

In this study, special attention was paid to the topographic relationships between the 3rd cerebral ventricle, on the one hand, neurons and nerve fibers immunopositive for DA-synthesizing enzymes, on the other. Thus, neurons, immunopositive for one or both DA-synthesizing enzymes, were found in the subependymal zone in the PeVN of rats on E18, E21, and P5. Nerve fibers with the same immunopositivity were found not only in the subependymal zone, but also between ependymal cells, as well as in the lumen of the 3rd cerebral ventricle. The latter appears to be more common in rats on P5 than on E21 (Figure 8).

## 3. Discussion

Despite the fact that the PeVN has long been considered only as a hypothalamic center for the regulation of reproduction by the neurohormone kisspeptin [33,34], it has only recently been shown that, at the same time, the PeVN in adulthood is one of the largest so-called mixed DAergic centers containing DAergic neurons and neurons expressing one of the DA-synthesizing enzymes [11]. According to previous studies, neurons of this kind secrete L-DOPA and DA, which can play the role of neurotransmitters in the brain itself or possibly neurohormones when delivered to the cerebrospinal fluid (CSF) and the blood vessels [11,23,24,25,32]. Given that both substances are potential morphogenetic (transcriptional) factors [29,30,31], it is of undoubted interest to study the development of the PeVN as a mixed DAergic center and a source of L-DOPA and DA. This has been the impetus for this pioneering study.

**Figure 9:**
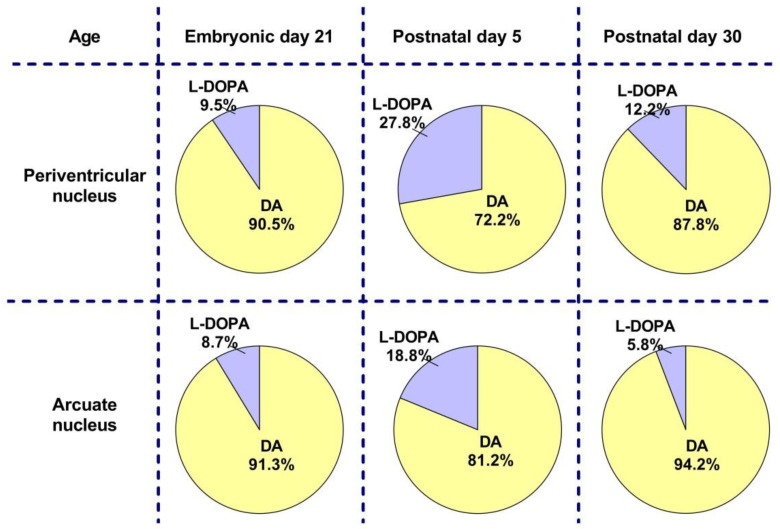
Comparative analysis of the proportion of dopamine (DA) and L-DOPA as a percentage of their total content, taken as 100%, in the periventricular and arcuate nuclei on embryonic day 21 and on postnatal days 5 and 30 according to the data obtained in this and our previous studies [11].

First of all, it was necessary to check whether the PeVN is the same powerful source of L-DOPA and DA in the perinatal period, a critical period of brain morphogenesis, as in adulthood [11]. To do this, we have compared the content of L-DOPA and DA in the PeVN and in the AN, another already well-studied mixed DAergic center of the brain [11,22]. Our study showed that L-DOPA and DA are undetectable in the PeVN on E18, whereas at the end of the prenatal period (on E21), in the early postnatal period (on P5) (see Results), and in adulthood [11], the content of both substances in the PeVN is comparable to their content in the AN (Figure 9). When the total content of L-DOPA and DA is taken as 100%, then in both nuclei the maximum proportion of L-DOPA (27.8%) falls on P5 (Figure 9). Based on this fact, it can be assumed that L-DOPA at this stage of ontogenesis is more in demand by the brain as a morphogenetic factor than DA. No less interesting is the fact that the DA content in the AN on E21, on P5, and especially on P30 significantly exceeds the content of DA over the same periods in the PeVN (Figure 9). These data suggest that there is a greater demand for DA in the AN compared with the PeVN throughout life, which is probably due to the fact that DA is released in large amounts into the pituitary portal circulation, providing inhibitory control of pituitary prolactin secretion as a neurohormone [23,32].

**Figure 10:**
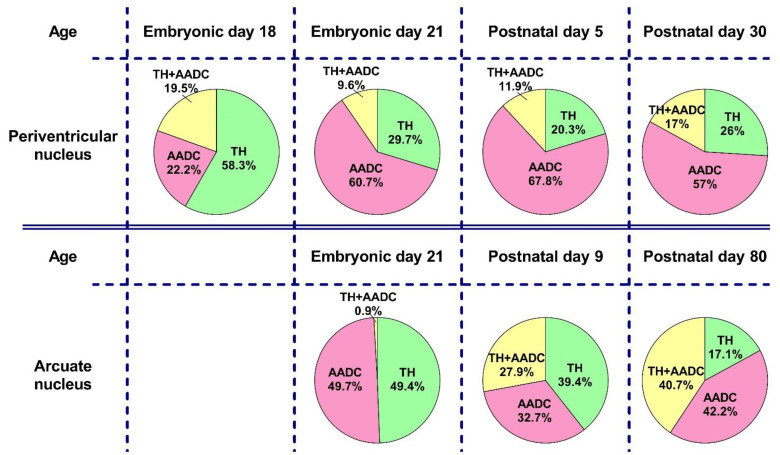
Comparative analysis of the proportion of neurons expressing tyrosine hydroxylase (TH) and aromatic L-amino acid decarboxylase (AADC), as well as bienzymatic neurons (TH + AADC), as a percentage of their total number, taken as 100%, in the periventricular and arcuate nuclei on embryonic days 18 and 21 and on postnatal days 5, 9, 30 and 80, according to the data obtained in this and our previous studies [11,26].

Based on the above data on the content of L-DOPA and DA in the PeVN in rats during the perinatal period, we assumed that, as in adulthood, the developing PeVN contains monoenzymatic TH-expressing neurons that produce L-DOPA, as well as monoenzymatic AADC-expressing neurons and DAergic (bienzymatic) neurons synthesizing DA from L-DOPA and L-tyrosine, respectively. This assumption has been supported by the results of our morphological study using double immunolabeling for TH and AADC. Indeed, in rats in all the studied age groups (on E18, E21, and P5), we found all three types of neurons: those expressing only TH, expressing only AADC, and expressing TH and AADC (Figure 10). Although all these neurons were present in fetuses on E18, at this age their number was extremely small, it did not exceed 50.

From a functional point of view, an important manifestation of the PeVN development was a change in the ratio of the number of monoenzymatic neurons and bienzymatic neurons. This ratio in the studied age groups was as follows: 4.2 for E18, 9.4 for E21, 7.4 for P5 (see Results), and 4.8 for P30 [11] (Figure 10). This suggests that the maximum need of the brain and body for the presence of monoenzymatic neurons and for their specific syntheses falls on the perinatal period: to a greater extent on E21 and to a somewhat lesser extent on P5. These data correlate well with those of our previous study on the development of the AN [26]. This study showed that in rats, until the end of the intrauterine development, the AN contains numerous monoenzymatic neurons, but almost no bienzymatic neurons. In the postnatal period, bienzymatic neurons appear in the AN, and their number increases with age. However, even in adult rats, their proportion does not exceed 50% of all neurons expressing enzymes of DA synthesis [11,26].

Interestingly, the proportion of monoenzymatic TH-expressing neurons in the PeVN changes little during ontogenesis, whereas the number of these neurons increases significantly and approximately equally in each age interval: by 330 neurons from E18 to E21, and by 350 neurons from E21 to P5 (Figure 10). A much smaller increase in these neurons was also noted in the interval from P5 to P30, by only 80 neurons. These data can be considered, although with caution, as an additional argument in favor of our hypothesis about the particularly high need of the brain and the body to increase L-DOPA synthesis in the critical period of brain morphogenesis. The same trend, but much more pronounced, is characteristic of changes in the number of monoenzymatic neurons expressing AADC. A sharp increase in the number of these neurons was shown in the interval from E18 to P5, followed by a decrease by P30 [11] (Figure 10), which correlates with changes in the DA content in the PeVN over the same period of ontogenesis. Taken together, both facts can be considered as an indicator of an increased need for DA in the brain, which is synthesized not only by DAergic neurons from L-tyrosine, but also by monoenzymatic AADC-neurons from extracellular L-DOPA. In other words, these data suggest an increased synthesis of DA in the PeVN during the critical period of brain morphogenesis, supporting the idea that DA at this time is involved in the regulation of brain development as a morphogenetic (transcriptional) factor [29,31]. Despite the attractiveness of our hypothesis that L-DOPA and DA, synthesized in the PeVN, play the role of morphogenetic factors in the perinatal period, direct evidence must be obtained in the future.

When evaluating the chemical machinery for the synthesis of L-DOPA and DA in the PeVN in ontogenesis, one cannot ignore the fact that in a number of mixed DAergic centers, for example, in the AN and in the striatum, DA is synthesized by monoenzymatic neurons expressing only TH or only AADC in cooperation [20,22]. In this case, L-DOPA, secreted by monoenzymatic TH neurons, diffuses along the intercellular clefts to the nearest monoenzymatic AADC neurons, where DA is synthesized [20,22]. In our previous study, it was shown that DA is not synthesized by monoenzymatic neurons in the PeVN in adult rats [11], which, however, does not exclude such a possibility at earlier stages of ontogenesis. According to our methodology, evidence for the presence of cooperative DA synthesis is based on demonstrating a decrease in DA synthesis in sections of the brain region of interest in the presence of BCH, an inhibitor of large L-amino acid transporter type 1, a membrane transporter of large neutral L-amino acids and L-DOPA to neurons, compared with the control, when brain sections are incubated in a medium without BCH [20]. In this study in rats on P5, as in our previous study on P30 [11], no cooperative synthesis of DA was observed. This does not exclude the fact that monoenzymatic AADC-neurons can synthesize dopamine using L-DOPA delivered from the cerebrospinal fluid and blood to the nervous tissue [11].

In order to fully understand the functional role of L-DOPA and DA synthesized in the PeVN, it was first necessary to establish where these substances are transported along the axons at different stages of ontogenesis. For this purpose, we used confocal microscopy in combination with double immunolabeling of neurons and nerve fibers for DA-synthesizing enzymes. When studying the projections of the monoenzymatic and bienzymatic nerve fibers in the PeVN, we obtained qualitatively identical results in all age groups of animals, which generally coincided with the recently obtained results for the PeVN in adult rats [11]. Indeed, we have shown close topographic relationships between all types of neurons and nerve fibers, which suggests the participation of L-DOPA and DA synthesized in these neurons in intercellular signaling.

Of particular interest are our confocal microscope observations of numerous nerve fibers of all the described types in terms of the content of DA-synthesizing enzymes, which are located close to the 3rd ventricle: in the subependymal zone, between the ependymal cells and supraependymally—in the lumen of the ventricle. These data suggest sprouting of axons of neurons expressing DA-synthesizing enzymes in the 3rd ventricle and the release of L-DOPA and DA into the CSF. Previously, we have shown that the CSF in developing rats contains the main monoamine neurotransmitters including DA [35]. We believe that these neurotransmitters, on the one hand, are delivered to the CSF from brain neurons, and, on the other hand, can diffuse from the CSF to the brain, participating in neuroendocrine regulation or volume neurotransmission of target neurons located in the periventricular region of the brain. Of particular interest is the coincidence in time of the maximum content of DA in the PeVN and the maximum concentration of DA in the CSF—in rats at P5 [35].

Thus, we have shown for the first time that the PeVN as a mixed DAergic center develops in the perinatal period of ontogenesis, being a powerful source of L-DOPA and DA, which are presumably involved in the regulation of brain development.

## 4. Materials and Methods

### 4.1. Animals

**Figure 11:**
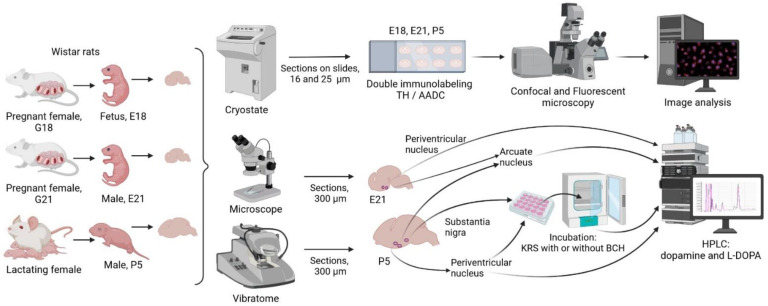
The design of experiments on Wistar rats on embryonic day 18 (E18), E21, and on postnatal day 5 (P5). AADC, aromatic l-amino acid decarboxylase; BCH, 2-aminobicyclo[2.2.1]heptane-2-carboxylic acid; L-DOPA, L-3,4-dihydroxyphenylalanine; HPLC, high-performance liquid chromatography; KRS, Krebs-Ringer solution; TH, tyrosine hydroxylase.

In this study, Wistar rats were used: females on the 18th (*n* = 6) and 21st (*n* = 9) days of pregnancy, fetuses on embryonic day 18 (E18) without sex differentiation (*n* = 38), males on E21 (*n* = 40), and males on postnatal day 5 (P5) (*n* = 56) weighing 8–10 g (Figure 11). The day of detection of spermatozoa in a smear was considered the 1st day of pregnancy and, accordingly, E1, and the day of birth was considered the first day of the postnatal period, P1. The animals were kept under standard vivarium laboratory conditions: at a temperature of +21–23 °C, with a 12 h day-night regimen, and with free access to food and water.

All manipulations with the animals were performed in accordance with the requirements of the NIH Guide for the Care and Use of Laboratory Animals and the Bioethics Committee of the Koltzov Institute of Developmental Biology of the Russian Academy of Sciences (protocol No. 44 dated 24 December 2020 and protocol No. 55 dated 9 December 2021).

### 4.2. Experiments and Obtaining Samples for Analysis

#### 4.2.1. Obtaining Periventricular and Arcuate Nuclei Samples for Biochemical Analysis

Rats on the 18th (*n* = 3) and 21st (*n* = 6) days of pregnancy were anesthetized with isoflurane (Laboratorios Karizoo, Barcelona, Spain) using the SomnoSuite anesthesia machine (Kent Scientific, Torrington, CT, USA). Laparotomy was then performed, and the fetuses were removed from the uterus. The sex of the fetuses was determined on E21. The fetuses on E18 (*n* = 30) and male fetuses on E21 (*n* = 32), as well as male rat pups on P5 (*n* = 16) were then decapitated, and their brains were removed.

From the isolated brains in artificial cerebrospinal fluid solution (mM: NaCl 119, KCl 2.5, NaH2PO_4_ 1.2, NaHCO_3_ 25, CaCl_2_ 2, MgSO_4_ 2, d-glucose 12.5, ascorbic acid 0.5; pH 7.3–7.4) saturated with carbogen (O_2_: 95%, CO_2_: 5%), frontal sections were prepared under dissecting microscope on E18 and E21—300 µm thickness, and using a vibratome (Leica VT 1200S, Leica, Wetzlar, Germany) on P5—900 µm thick. The PeVN and the AN were excised from the sections according to Atlas of the developing rat nervous system [36] (Figure S1), adjusted for age. In the dorsoventral direction, the PeVN was isolated at the level of the middle third of the 3rd ventricle, and the AN was isolated at the level of the lower third of the 3rd ventricle (Figure S1). As one sample for analysis, we used PeVN and AN tissue cut from vibratome sections of both hemispheres of the brain: on E18 from 10 fetuses, on E21 from 8 fetuses, and on P5 from 2 pups. The resulting tissue samples were weighed, frozen in liquid nitrogen, and stored at −70 °C until DA and L-DOPA were determined using high performance liquid chromatography.

#### 4.2.2. Incubation of Vibratome Sections of the Periventricular Nucleus and Substantia Nigra of Rats on Postnatal Day 5

Male rats on P5 (*n* = 32) were anesthetized with isoflurane as described in Section 2.2 and decapitated, and their brains were isolated. Serial 300 µm thick frontal sections were then prepared on a vibratome in KRS (mM: NaCl 120, KCl 4.8, CaCl_2_ 2, MgSO_4_ 1.3, NaHCO_3_ 25, d-glucose 10, HEPES 20, ascorbic acid 0.1, pH 7.4) at 4 °C. Then, 3 frontal sections were taken from each brain at the level of the PeVN or the SN (Figure 2), and under the control of a dissecting microscope, PeVN or SN tissue was cut with a blade. The resulting sections were divided into two halves, symmetrical with respect to the midsagittal plane. For incubation, 6 halves of PeVN or SN sections from 2 rats were taken into each chamber. PeVN and SN sections were placed in separate thermostatic (+37 °C) chambers with a volume of 1.5 mL each (20 chambers per brain region) and were incubated in 1000 µL KRS at 37 °C for 30 min to stabilize the tissue. Then, in half of the chambers (10 chambers per brain region) with PeVN and SN sections, KRS was replaced with KRS containing 0.5 mM BCH, an inhibitor of L-amino acid transporter 1 [37]. 1 μM NH_4_OH was used to dissolve BCN, according to the manufacturer’s recommendation (Catalog A7902, Sigma, Saint Louis, MO, USA). The vibratome sections were incubated for 1 h at +37 °C, during which the incubation medium was collected and replaced with a fresh medium every 20 min, three times in total. In the control (10 chambers per brain region), PeVN or SN sections obtained from different cerebral hemispheres were incubated. In this case, the sections were incubated in KRS without BCH but with 1 µM NH_4_OH. After the end of the incubation, the sections were weighed, frozen in liquid nitrogen, and stored at −70 °C until measuring DA and L-DOPA. 100 µL 1 N HClO_4_ and 2 pmol/mL 3,4-dihydroxybenzylamine hydrobromide were added to the fractions of the incubation medium obtained after the incubation of PeVN sections, and 100 µL 1 N HClO_4_ and 5 pmol/mL 3,4-dihydroxybenzylamine hydrobromide were added to the fractions of the incubation medium obtained after the incubation of SN sections. The resulting samples were frozen in liquid nitrogen and stored at −70 °C until measuring DA and L-DOPA.

#### 4.2.3. Preparing the Brain for Immunohistochemistry

In rats on the 18th and 21st days of pregnancy under chloral hydrate anesthesia (i.p. 400 mg/kg), the fetuses were extracted. Rats at P5 were also anesthetized with chloral hydrate. Rats at E18, E21, and P5 (*n* = 7) were perfused sequentially through the heart with the following: (a) 0.9% NaCl in 0.02 M phosphate buffer (phosphate-buffered saline, PBS) (pH 7.2–7.4) at +37 °C; on E18 and E21, for 2 min, and on P5, for 10 min; (b) 4% paraformaldehyde in 0.1 M phosphate buffer (pH 7.2–7.4) at +4 °C; on E18 and E21, for 2 min, and on P5, for 10 min. After that, the animals were decapitated and their brains were removed, postfixed by immersion in 4% paraformaldehyde for 12 h at +4 °C, and then washed in PBS. After fixation, the brains were incubated in 20% sucrose in 0.02 M PBS at +4 °C for 24 h, frozen in hexane at −40 °C, and stored at −70 °C until immunocytochemistry was performed.

### 4.3. Methods

#### 4.3.1. High Performance Liquid Chromatography

Frozen nervous tissue samples were thawed. For E18 and E21, 4 tissue samples were then combined into one sample, and for P5, one tissue sample was taken as a single sample. All samples were homogenized in 120 μL 0.1 N HClO_4_ with 3,4-dihydroxybenzylamine hydrobromide (5 pmol/mL) using an ultrasonic homogenizer (UP100H, Hielscher, Germany). The sample homogenates were then centrifuged for 15 min at 20,000 g and +4 °C and the supernatant was collected, in which DA and L-DOPA were measured.

High performance liquid chromatography separation was carried out on a reversed-phase column ReproSil-Pur, ODS-3, 4 × 100 mm with a pore diameter of 3 µm (Dr. Majsch GMBH, Entringen, Germany), at a temperature of +28 °C and a mobile phase speed of 1 mL/min, supported by a liquid chromatograph LC-20ADsp (Shimadzu, Kyoto, Japan) at 850 mV. Mobile phase consisted of 0.1 M citrate-phosphate buffer, 0.3 mM sodium octanesulfonate, 0.1 mM EDTA, and 8% acetonitrile (all reagents from Sigma, Saint Louis, MO, USA) (pH 2.58). DA and L-DOPA were determined using a fluorescent detector RF-20A (Shimadzu, Kyoto, Japan) at a wavelength of 285/316 nm. The peaks of the substances were identified by the time of their release into the standard solution.

#### 4.3.2. Immunohistochemistry

For immunostaining of neurons, frontal serial sections of PeVN, 16 µm or 25 µm thick, were prepared on E18, E21, and P5 using a cryostat (Leica CM1950, Leica, Wetzlar, Germany). All serial sections with a thickness of 16 µm were mounted on slides, and from serial sections with a thickness of 25 µm, every second section was mounted on slides on E18 and E21, and every third section on P5. 16 µm thick sections on slides were sequentially incubated in PBS containing: (a) 1% sodium lauryl sulfate (Sigma, Saint Louis, MO, USA), for 5 min at +20 °C; (b) 5% bovine serum albumin (Sigma, Saint Louis, MO, USA) and 0.3% Triton X-100 (Sigma, Saint Louis, MO, USA), for 1 h at +20 °C; (c) mouse monoclonal antibodies to TH (1:700) (Sigma, Saint Louis, MO, USA), rabbit polyclonal antibodies to AADC (1:300) (Abcam, Cambridge, UK), 1% bovine serum albumin, and 0.1% Triton X-100, for 20 h at +20 °C; (d) goat Alexa-Fluor-546 antibodies against rabbit gamma globulins (1:1000) (Invitrogen, Waltham, MA, USA), for 2 h at +20 °C; (e) donkey Alexa-Fluor-488 antibodies against mouse gamma globulins (1:1000) (Invitrogen, Waltham, MA, USA), for 2 h at +20 °C. After each incubation, except the last one, the sections were washed in PBS three times for a total of 45 min. After the last incubation, the sections were washed in PBS for an hour and placed into a medium containing 4′,6-diamidino-2-phenylindole (Abcam, Cambridge, UK), a dye for cell nuclei.

Then, 25 µm thick PeVN sections on slides were sequentially incubated in PBS containing: (a) 1% sodium lauryl sulfate (Sigma, Saint Louis, MO, USA), for 5 min at +20 °C; (b) 5% bovine serum albumin (Sigma, Saint Louis, MO, USA) and 0.3% Triton X-100 (Sigma, Saint Louis, MO, USA), for 1 h at +20 °C; (c) mouse monoclonal antibodies to TH (1:700) (Sigma, Saint Louis, MO, USA), rabbit polyclonal antibodies to AADC (1:300) (Abcam, Cambridge, UK), 1% bovine serum albumin, and 0.1% Triton X-100, for 20 h at +20 °C; (d) biotinylated goat antibodies against rabbit gamma globulins (1:200) (Vector Laboratories, Newark, CA, USA), for 2 h at +20 °C; (e) streptavidin-Cy3 (1:100) (Sigma, Saint Louis, MO, USA), for 1 h at +20 °C; and (f) Alexa Fluor 488 donkey antibodies against mouse gamma globulins (1:1000) (Invitrogen, Waltham, MA, USA), for 2 h at +20 °C.

#### 4.3.3. Microscopy and Quantitative Image Analysis

After double immunostaining for TH and AADC, 16 µm thick sections containing PeVN were examined using a fluorescence microscope Zeiss Observer Z1 (Zeiss, Oberkochen, Germany) with a 20× objective. To create a panoramic image, the “mosaic” function (AxioVision 4.8 software, Zeiss, Oberkochen, Germany) was used, so that the entire 3rd ventricle and 300 μm area of the PeVN on each side of the ventricle were visible on the final image. Next, we counted the number of neurons immunopositive for TH or AADC, or for both enzymes, as well as the total number of these neurons located in the PeVN. Next, in the selected area, the number of neurons with 4′,6-diamidino-2-phenylindole-stained nuclei was counted. The Abercrombie test [38] was used to exclude double counting of neurons located on neighboring sections:

$$N=\frac{n*\Delta }{\Delta +d}$$

*N*—the total number of cells in a section

*n*—the number of counted cells in a section

Δ—section thickness (16 μm)

*d*—average cell nucleus diameter

In addition to the number of neurons that are TH-immunopositive only, AADC-immunopositive only, or both TH-immunopositive and AADC-immunopositive, we determined the proportion of each of these populations as a percentage of the total number of all neurons, taken as 100%, using the following formula:

$$P_{n}=\frac{N_{n}}{N_{TH}+N_{AADC}+N_{TH/AADC}}\times 100\%$$

*P_n_*—percentage of neurons that are TH-immunopositive only, AADC-immunopositive only, or both TH-immunopositive and AADC-immunopositive

*N_n_*—number of neurons that are TH-immunopositive only, AADC-immunopositive only, or both TH-immunopositive and AADC-immunopositive

*N_TH_*—number of TH-immunopositive neurons

*N_AADC_*—number of AADC-immunopositive neurons

*N_TH/AADC_*—number of neurons that are both TH-immunopositive and AADC-immunopositive

To assess the distribution of neurons in the rostrocaudal direction, the PeVN was divided into 5 segments. On E18, the length of each segment was 64 µm (4 sections); on E21, it was 80 µm (5 sections); and on P5, the length was 128 µm (8 sections). In each PeVN segment, the number of neurons with a stained nucleus, immunopositive for one or both enzymes, was counted.

#### 4.3.4. Confocal Microscopy and Image Analysis

After double immunolabeling for TH and AADC, brain sections on E21 and P5 containing PeVN were examined with a confocal microscope Zeiss LSM880 (Carl Zeiss, Jena, Germany) using the objectives Plan-Apochromat 20×/0.8 M2 and Plan-Apochromat 63×/1.40 Oil DIC M27. Photographs of the sections were taken in two channels. In the first channel, the signal from AlexaFluor 488 was detected, and in the second channel, those from Cy3 and 4′,6-diamidino-2-phenylindole. A panoramic shot was taken using the “mosaic” function 1 × 4 with z-stack (optical section thickness 1.3 μm) at a 20× magnification with an additional 0.6× zoom. To assess the contact between the neurons and/or nerve fibers, a z-stack (optical section thickness 0.5 μm) was made at a magnification of 63× with an additional 2× zoom. Image processing was done in ZenBlue (Carl Zeiss, Germany).

### 4.4. Statistical Analysis

Statistical analysis was performed using GraphPad Prism 6 software (GraphPad Software, La Jolla, CA, USA). The groups were compared for normality by using the D’Agostino and Pearson test. We used the paired t-test to assess the content of DA and L-DOPA in freshly prepared nervous tissue, whereas the Friedman test and Dunn’s post hoc test were used to assess the content of DA and L-DOPA in vibratome sections and in the incubation medium after incubating the sections. We used the non-parametric Mann–Whitney U-test to compare the results of IHC analysis between different types of neurons, whereas the Friedman test with Dunn’s post hoc test to assess the distribution of neurons of each type between zones. The results are presented as mean ± SEM or as median with interquartile range. Differences were considered significant at *p* < 0.05.

## 5. Conclusions

This study continues our long-term research on the so-called mixed dopaminergic centers of the brain, which contain both numerous DAergic neurons with two DA-synthesizing enzymes, and no less numerous neurons containing only one of the enzymes: AADC or TH. The end secretory product of DAergic and monoenzymatic AADC neurons is DA, while monoenzymatic TH neurons produce L-DOPA. In adulthood, these substances play the role of neurotransmitters or neurohormones, whereas in the perinatal period, they can have an irreversible morphogenetic action on the developing brain. The latter circumstance necessitates a thorough study of the development of mixed DAergic centers of the brain in the perinatal period. Therefore, the aim of this study was to evaluate the development of the PeVN as one of the mixed DAergic centers. It has been shown that during the perinatal period, the total number of neurons containing DA-synthesizing enzymes triples, mainly due to monoenzymatic neurons; the content of L-DOPA, the end product of monoenzymatic TH neurons, doubles; and the content of DA, the end product of monoenzymatic AADC neurons and DAergic neurons, increases sixfold. From a functional point of view, it is important to note that these neurons are topographically closely connected with each other, as well as with the 3rd cerebral ventricle: this is manifested in confocal microscopy by the presence of numerous interneuronal and neuroventricular contacts. Overall, the data obtained show that the PeVN as a mixed DAergic center is developed in the perinatal critical period of brain development and suggest that L-DOPA and DA synthesized by monoenzymatic and bienzymatic neurons participate in the regulation of brain development as paracrine and neuroendocrine morphogenetic factors.

## Data Availability

The data presented in this study are available on request from the corresponding author. The data are not publicly available due to legal issues.

## Supplementary Materials

The following supporting information can be downloaded at: https://www.mdpi.com/article/10.3390/ijms232314682/s1, Figure S1: Scheme of fetal and neonatal rat brain regions.

## Abbreviations

AADC	aromatic L-amino acid decarboxylase
AN	arcuate nucleus
BCH	2-aminobicyclo[2.2.1]heptane-2-carboxylic acid
DA	dopamine
E	embryonic day
KRS	Krebs-Ringer solution
L-DOPA	L-3,4-dihydroxyphenylalanine
P	postnatal day
PBS	phosphate-buffered saline
PeVN	periventricular nucleus
SN	substantia nigra
TH	tyrosine hydroxylase
