# Supplementary figures and images for: Development of the Periventricular Nucleus as a Brain Center, Containing Dopaminergic Neurons and Neurons Expressing Individual Enzymes of Dopamine Synthesis
Source: Int J Mol Sci. 2022 Nov 24;23(23):14682. doi: 10.3390/ijms232314682 (PMC9736787; doi:10.3390/ijms232314682)

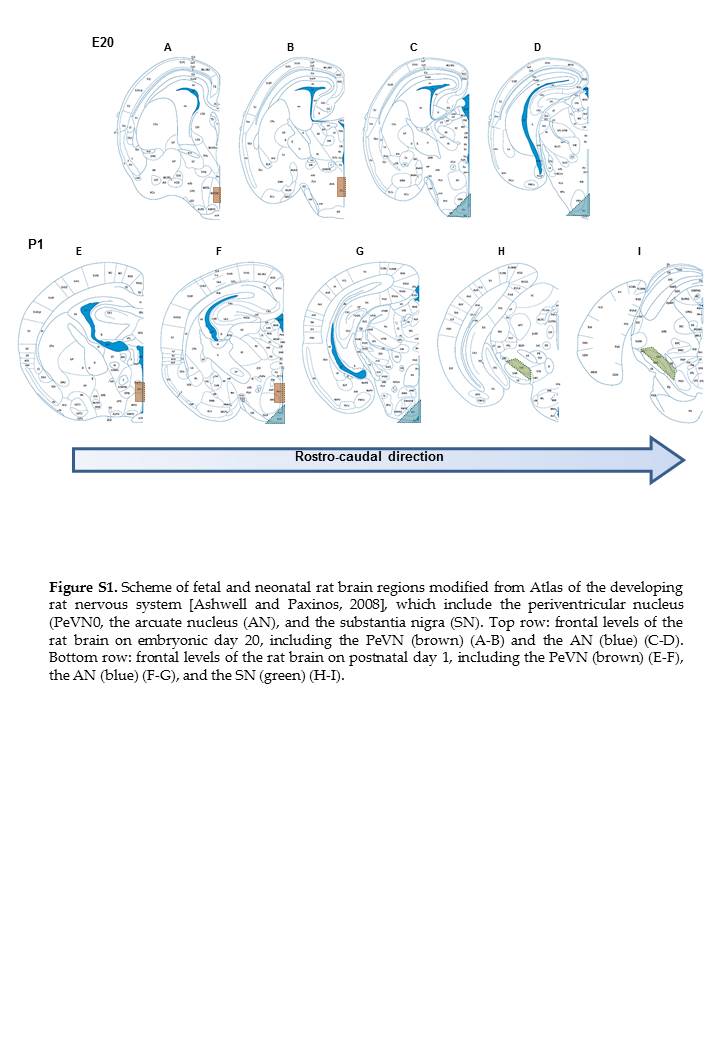

Supplement: Supplementary file 1 [file ijms-23-14682-s001.zip › Supplementary_Figure_S1.jpg]
